# Scarlet Hose

**Published:** 1870-01

**Authors:** 


### SCARLET HOSE.

THE cry against scarlet hose and mauve shirts is not a false alarm; and it it to be presumed that the gentleman who had been advertising for information from "all persons who have suffered from wearing colored socks, or other colored surface clothing," have good reason for prosecuting their inquiries. Of the poisonous character of some of the dyes used for these articles there can be no doubt. A French chemist has been investigating the point, and has brought the subject before the Paris Academy of Sciences. Picture to yourself a grave assembly engrossod with a discussion upon *les bas de soie rouge!* Blue-stockings in council upon red stockings! The doctor, Prof. Tardieu, had been consulted by a young man whose feet had been inflammed and ulcerated from the wearing of red socks; and at about the sane time, some other cases came before him of like evils, evidently traceable to a like cause. So he took his patients' *chaussettes*, and extracted the coloring from them by chemical treatment. Then he injected small quantities of it beneath the thigh-skins of a frog, a rabbit, and a dog. All the animals died with poisonous symptoms; the frog in four hours, the dog in thirty-six, and the rabbit on the third day after the experiment. Next he procured some of the original dye, known, from its beautiful tint, as coralline, and made similar trials with the same results. A colleague actually succeeded in dyeing a skein of silk with the matter re-extracted from the lungs and liver of one of the poisoned subjects. Coralline is a near relative of aniline, the blue and violet dyes from which, are stated by an able chemist, to be dangerously contaminated with arsenic. It is a teacherous family altogether, and we must be chary of allowing our connection with it to become too intimate.

SPLENDID JOURNALS.—Elmira is becoming celebrated for her newspapers. The handsomest and best edited Journals along the line of the Erie Road, are printed in this city. The *Advertiser* intends fitting up handsome apartments in the Hathaway House, using the dining room for the compositors' room—the old office will be used for the business office—while several elegant editorial rooms will be fitted up on the same floor. The basement will be occupied by the engine and heavy presses. When this arrangement is completed we expect to see one of the best appointed printing concerns in the country. The *Advertiser* will also enlarge and greatly improve its appearance—will appear in a bran-fired-new-dress, with an increased editorial corps. Ishmael, Mr. Towner, will be called to one of the editorial chairs, while Robt. M. Watts will take charge of the Job Department. Look out for a splendid *Advertiser* on first of January.

*The Gazette* has also been beautifying its appearance and enlarging its facilities. It always has been a favorite family paper and always will be as long as Charley Hazard runs the concern.

*The Review* has passed into the hands of Mr. Wheeler, an old and experienced printer. It has an able corps of editors, and is the handsomest weekly in the State.

*The Bistoury*—speaks for itself—we are proud of it, and hope its patrons are also.

Altogether, Elmira has reason to be proud of her Journals—no city in the State (outside the metropolis,) can exhibit better ones.

